# Interactions between Oral Tissues and External Light and Matters

**DOI:** 10.1155/2012/726259

**Published:** 2012-12-26

**Authors:** S. Nammour, H. S. Loh, R. De Moor, C. P. Eduardo

**Affiliations:** ^1^Department of Dental Sciences, Faculty of Medicine, University of Liege, Liege, Belgium; ^2^Department of Oral and Maxillofacial Surgery, Faculty of Dentistry, National University Hospital, Singapore; ^3^Department of Operative Dentistry and Endodontology, Dental School, Gent University, Belgium; ^4^Laboratory of Lasers in Dentistry (LELO), Department of Restorative Dentistry, School of Dentistry, University of Sao Paulo, Brazil

We are currently in an era where the international scientific publication is highly valued, acting as a strong parameter for evaluation mechanism in ranking universities in the competitive scientific world. In this context, writing the editorial of this special issue gives us a great responsibility. 

After a cautious revision of the several articles submitted for publication in this special issue by the Editorial Board of this journal, the accepted papers translate the commitment of the authors to the scientific research. 

This special issue is a sample of the current research efforts addressing issues related to the interaction between the light and the oral tissues, highlighting subjects such as the treatment of pathologies associated with high intensity laser for better healing and welding, the use of high intensity laser treatment for cosmetic dentistry, evaluation of adhesive systems and dentin hypersensitivity, the detection and treatment decision of caries lesions with laser fluorescence, the treatment of root perforation using high intensity laser, and also the low level laser therapy for improving wound healing.

We hope that the readers can enjoy this special issue and improve their knowledge about the light interaction with the hard and soft oral tissues.

*S. Nammour* *S. Nammour* *H. S. Loh* *H. S. Loh* *R. De Moor* *R. De Moor* *C. P. Eduardo* *C. P. Eduardo*

